# The Fear of Losing—Nonsuicidal Self-Injury as a Protective Mechanism in Eating Disorders

**DOI:** 10.3389/fpsyt.2019.00825

**Published:** 2019-11-15

**Authors:** Eran Sagiv, Gergö Hadlaczky, Noga Sheetrit, Eitan Gur, Netta Horesh, Yari Gvion

**Affiliations:** ^1^Department of Psychology, Bar-Ilan University, Ramat Gan, Israel; ^2^Department of Learning, Informatics, Management and Ethics, Karolinska Institutet, Stockholm, Sweden; ^3^The Eating Disorders Department, Sheba Medical Center, Ramat Gan, Israel; ^4^Department of Psychology, Academic College Tel Aviv–Yaffo, Yaffo, Israel

**Keywords:** eating disorder, anorexia, loss-aversion, impulsivity, nonsuicidal self-injury, suicidal behavior

## Abstract

**Background:** This study examined the moderating role of loss aversion (LA) on the relationship between impulsivity, nonsuicidal self-injury (NSSI), suicidal attempts, and ideations among Eating Disorder (ED) patients.

**Methods:** Data was collected on 81 ED patients and 37 healthy controls. ED patients were divided into 2 groups: 25 AN-Rs, 56 AN-BPs and BNs. Measurements of trait impulsivity, LA, NSSI, suicide attempts, and suicide ideations were collected.

**Results:** The rate of attempting suicide was highest in the AN-BP/BN (34.8%), lower in the AN-Rs (8%), and the lowest in the controls (2.7%). Suicide ideation was also higher in AN-BP/BN compared to both AN-R and controls. NSSI was higher in the AN-BP/BN group compared to both AN-R and control groups. LA scores were lower among participants with EDs compared to controls. BMI and depression were positively associated with suicide ideation and NSSI. Impulsivity was associated to suicide attempt and suicide ideation. Contrary to our hypothesis, LA scores were positively correlated with NSSI and SI. A stepwise regression revealed that contradictory to our hypothesis, higher LA predicted NSSI prevalence severity of NSSI and suicide ideation.

**Limitations:** (1) Cross-sectional design; (2) Relatively small sample size of clinical subjects and only female participants; (3) Heterogeneity of treatment status.

**Conclusions:** EDs are associated with lower levels of LA compared to general population. Although high LA is considered a protective factor against “high damage” decisions, it may serve as a facilitator of lower risk decisions which help the individual soothe and communicate his or her own suffering such as NSSI.

## Introduction

Eating disorders (EDs) are a serious public health problem with long-term effects on physical and mental health (1). Its prevalence ranges between 2–4% (2). Self-harm behaviors, including nonsuicidal self-injury (NSSI) (3), completed suicide (4, 5), and suicide attempts (6, 7) are considered common in individuals with EDs.

Anorexia nervosa (AN) of the restrictive (AN-R) and binge–purge subtypes (AN-BP), and bulimia nervosa (BN) have common neurobiological mechanisms (8, 9). Yet challenges arise when attempting to discriminate between AN and BN using contemporary classification methods (i.e., Diagnostic and Statistical Manual of Mental Disorders, 5th edition; International Statistical Classification of Diseases and Related Health Problems, 10th revision), which rely on observable symptoms [e.g., body mass index (BMI)] and behaviors (10, 11), due to similarities in these diagnoses. While an AN-BP patient may be much closer in nature to a BN patient than a AN-R patient, he or she may be given the same diagnosis as an AN-R patient. The need to find a more effective way to diagnose and differentiate between AN and BN patients accords with the novel approach proposed by the United States National Institute of Mental Health, which aims to find a framework beyond categorical and symptom-based approaches (12–14).

Studies on impulsivity and EDs have suggested impulsivity as a potential transdiagnostic concept (15, 16) that may facilitate a better understanding of psychological profiles in EDs. Impulsivity can be conceptualized as either a stable state or a dynamic-temporal state (17–19). Several studies have found impulsivity to be a key behavioral characteristic among individuals with EDs, in general (20–23), and particularly in AN-BP and BN patients who report higher percentages of impulsive behavior compared to AN-R patients (24, 25, 15, 26). Moreover, studies show that patients who exhibit binge–purge behaviors are more inclined to present other impulsive behaviors as well as emotional swings and emotional disturbances compared with those who do not exhibit binge–purge behaviors (27, 28). Impulsive behaviors such as bingeing and purging are often attributed to an escape mechanism adopted to evade an unpleasant situation (29). Various studies have shown impulsivity to be a major risk factor in carrying out NSSIs and suicide attempts (30, 31).

NSSI is defined as self-inflicted harm to one’s body (e.g., cutting, incising, burning) without the intent of suicide (32). NSSI is associated with a history of childhood abuse, emotional overreaction, difficulty in problem solving, and communication difficulties. It is considered a coping strategy to gain control over impulses, affect regulation, interpersonal communication when previous attempts have failed, and self-punishment resulting from self-criticism or externalization (33). A functional approach suggests that self-harm is sustained by negative intrapersonal reinforcement (avoiding and deflecting emotions and negative thoughts), positive intrapersonal reinforcement (producing desired emotions or stimuli), negative interpersonal reinforcement (escaping and avoiding undesirable social situations), and positive interpersonal reinforcement (eases the ability to ask for help) (33, 34).

A recent meta-analysis found that 27.3% of individuals with an ED had a history of NSSI. Among those suffering from AN, prevalence of NSSI was 21.8%, while among those suffering from BN, the prevalence was 32.7% (3). Among ED patients who exhibit binge or purge behavior, there was a significantly higher number of self-injury symptoms than other ED patients (35). Research also suggests that self-harm is correlated with self-reporting impulsivity (36). Among ED patients, 38.9% reported at least one type of NSSI (37).

NSSI is closely related to suicidal behavior, which is defined as an act of intentionally ending one’s own life (38, 39). Suicide may be preceded by suicidal ideation, threats, and gestures, nonsuicidal self-injuries, and suicide attempts with various degrees of lethality (40). According to the National Association of Anorexia Nervosa and Associated Disorders, EDs have the highest mortality rate of any mental disorder. Given that both suicidal behaviors and EDs are body-focused disorders, over the past decade, many studies have turned to investigate their co-occurrence (30).

While personality assessment tools can help characterize individuals who fit the impulsive trait profile, the existence of additional manifestations of impulsivity oblige clinicians to seek out alternative methods to define and measure impulsive tendencies and behaviors. The process underlying these attempts is studied through models of decision-making (41, 42). People suffering from AN or BN turn to food or refuse food intake for reasons that are unrelated to their nutritional needs. Such decisions can be attributed to faulty decision-making patterns. ED symptomatology comprises a variety of behaviors, such as bingeing, purging, fasting, or self-injury, that lead to immediate satisfaction despite their long-term adverse effects. These behaviors have been shown to be related to a specific component of decision-making, namely delayed discounting (43, 44), which describes the substantial overvaluation of immediate, as opposed to delayed, rewards. Another well-known bias that influences decision-making processes is loss aversion (LA). Human beings are driven by the avoidance of losses rather than by procuring equivalent gains. This universal behavioral pattern was described by (45, 46) who demonstrated that the subjective impact of losses appears to be roughly twice that of gains in human subjects. The pervasiveness of LA has been found in many empirical studies (47), and has been evaluated by having participants gamble a certain amount of money on the outcome (win or lose) of tasks they perform (e.g., 48, 49). The extent of potential gains and losses are then varied for each gamble, and participants are asked if they are willing to gamble.

The literature on the association between LA and EDs is very scarce. Moreover, to the best of our knowledge, a comparative study of LA in the different subtypes of AN and BN has yet to be conducted, although we can infer from previous studies that individuals with AN-R made less risky choices on the Balloon Analogue Risk Task compared with healthy controls (50). Chan et al. (51) found that individuals with BN show greater relative sensitivity to gains as opposed to losses on the Iowa Gambling Task, when compared to a group of AN patients and healthy controls. Research in the field of skin conductance responses in ED patients (52) has shown that prior to choosing cards on the Iowa Gambling Task, patients with AN had decreased anticipatory skin conductance responses compared to a group of recovered anorexics and a healthy control group, thereby suggesting that they were less anxious about aversive outcomes.

Although previous studies have made major contributions to the understanding of NSSI and suicidal behavior among ED patients, to the best of our knowledge, so far, no work has examined the association between decision-making processes and self-harm behaviors in ED patients, in general, and the moderating role of LA, in particular. Intuitively, it is reasonable to assume that individuals with a greater aversion to potential loss (e.g., body pain, long-term or irreversible damage to the body, scarring as a result of cutting, health risks, hospitalization, sorrow inflicted on family, etc.) would find the options of either NSSI and suicide less advantageous. On the other hand, in cases in which the potential gains of NSSI or suicide (ceasing pain, anxiety, and fear) are evaluated as higher than the potential losses, either act will be carried out. The clinical presentation of AN-R, as characterized by more rigidness and less impulsivity compared to AN-BP and BN, suggests that there may be an underlying cognitive bias of higher sensitivity to potential negative outcomes (losses) than positive outcomes. On the other hand, the clinical presentation of individuals with AN-BP and BN, as characterized by more impulsivity, suggests that there may be an underlying bias of a lesser sensitivity to potential negative, rather than positive, outcomes (losses).

In this study, we aimed to investigate the moderating role of LA in the association between impulsivity and self-harm behavior (NSSI and suicidal behavior) among ED patients. Consequently, we aimed to examine several hypotheses: first, we hypothesized that EDs of the AN-BP and BN groups will score higher on impulsivity, NSSI, and suicide attempts and ideation compared to AN-R and healthy controls. Second, we hypothesized that higher measures of LA will be displayed in the AN-R subjects compared to AN-BP and BN subjects, while healthy controls will display higher measures of LA compared to the research group. Third, we hypothesized a negative association between LA, NSSI, and suicide attempts among ED patients, and that the effect of LA will increase the risk of suicide attempts and NSSI among these patients, over and above the contribution of diagnosis, depression, BMI, and impulsivity.

## Method

### Study Sample

The study described in this report refers to patients with a current diagnosis of AN or BN being treated at the department for EDs in adults in a general hospital. A control group of students from Bar Ilan University was recruited. All 132 subjects were women who were divided into two groups: 93 clinical subjects and 39 healthy controls. Out of the EDs group, 12 were excluded from this study due to an irrelevant diagnosis or a failure to meet all the inclusion/exclusion criteria. ED patients were divided into two groups, the first group was made out of 25 subjects diagnosed with AN-R. Due to the previously mentioned similarities between the AN-BP and BN populations, and in line with recent literature (14), we combined 30 subjects diagnosed with AN-BP and 26 subjects diagnosed with BN into a single binge/purge group of 56 subjects. Four subjects out of the ED group and one subject out of the controls did not complete the behavioral task measuring LA due to administration difficulties.

### Measures

#### Diagnosis

The psychiatric diagnoses were given by clinicians who interviewed the subjects in an intake session to assess suitability for treatment. Diagnoses were based on Diagnostic and Statistical Manual of Mental Disorders, 5th edition, criteria and excluded comorbidity with other non-ED diagnoses.

#### Self-Rating Questionnaires

A variety of questionnaires were used to capture demographic information, traits, states, and suicide attempt characteristics.

#### Demographics

Information on age, country of birth, marital status, number of children, type of residence (urban/rural), years of education, employment status, psychiatric medication, psychotherapy treatment, and attention-deficit/hyperactivity disorder (ADHD) diagnosis was collected from the participants by self-report.

#### Suicide Attempts

Past suicide attempts and their severity were assessed by the Suicide Behaviors Questionnaire developed by Osman et al. (53). Composed of four items, each tapping a different dimension of suicidality, items are scored on a 5- to 7-point scale and are summed up to an aggregate score. Reliability scores of the original questionnaire range between 0.87–0.88 in adolescents and 0.76–0.87 in the adult population. The Suicide Behaviors Questionnaire instrument was translated to Hebrew for research purposes by way of double translation.

#### NSSI

A modified version of the Deliberate Self-Harm Inventory (54) was used to assess engagement in self-injurious behaviors. This self-report questionnaire addresses six different self-injury items: cutting of the body, self-burning, carving into the skin, preventing the healing of wounds, and banging the head against hard objects. An additional item assessed whether the injury was severe enough to require medical treatment. Items were followed by 0–3 Likert scales for rating the frequency of each behavior; ranging from 0 (“never”) to 3 (“five times or more”). The total score was calculated as a mean of the six items. The updated version of this instrument was translated into Hebrew [71]; in the current research reliability was found to be 0.83.

#### Depression

Depression was screened by using the depression module of the Patient Health Questionnaire. This questionnaire is based on DSM-IV criteria for major depressive disorder and includes nine items, each scored on a three-point scale (0 = ”not at all” to 3 = ”nearly every day”). Total scores of 5, 10, 15, and 20 have traditionally represented cut-oﬀ points for mild, moderate, moderate–severe, and severe depression, respectively. A score of 10 has been used as a cut-oﬀ point for an indication of a positive screening for clinical depression [73]. Values of reliability in previous research were found to be 0.89 and 0.84 and test–retest reliability was 0.84 [73]. In the current study, reliability was found to be 0.82.

#### Impulsivity

Impulsivity was measured by the Barratt Impulsiveness Scale (BIS-11: 55; BIS-11-A: 56) which is comprised of the scores of three separate constructs: attentional, motor, and nonplanning, and an additive score. The Hebrew BIS-11 has been found to have adequate reliability (a = 0.79, 57). In this study, reliability was found to be 0.85.

#### Loss Aversion

A behavioral procedure was used to assess LA based on the procedure introduced by Sokol-Hessner et al. (58). Each participant was exposed to a series of 90 trials in each of which they were instructed to choose either a risky or riskless option. The risky option constituted a gamble with a 50:50 chance of winning or losing a certain amount of money, whereas the riskless option was a choice of neither winning nor losing ($0). For example, a participant could be instructed to choose between a 50% chance of winning $40 (and 50% chance of winning nothing) or a guaranteed choice of $0. LA was estimated for each participant by calculating the mean loss/gain ratio for the accepted gambles in the entire task. The higher average ratio reflects a higher preference for a “lower but safer” (i.e. more averse to losses) choice, whereas a lower ratio reflects a tendency to ignore the potential losses and choose the gamble (i.e. less averse to losses).

### Procedure

The study was conducted in accordance with the 1989 revised Helsinki Declaration and received International Review Board approval from the medical center involved in the study. All participants in the clinical group were recruited during a 24-month period (March 2016–January 2018), were 18 years old or older, and had active ED diagnosed by a certified clinician. Patients were excluded from the study if they were suspected to be in a psychotic state, if they had a BMI lower than 12, due to a possible cognitive impairment, or if they had other cognitive impairments or language difficulties that did not allow for the independent completion of the study assignments. Candidates were identified by the medical staff to ensure their suitability for the study. They were approached by research assistants (M.A. level) who explained the aims and purposes of the project, and were asked for their written consent to participate. The interviews and questionnaires were each administered over the course of a hour-long session. If a patient demonstrated acute distress, the interviewer requested assistance from the medical staff. Participants in the healthy control group were students with neither psychiatric diagnoses nor histories of ED related behavior who volunteered to participate in the research as part of completing their graduate degree requirements.

### Data Analysis

Statistical analysis was performed using SPSS software, version 23. Group differences in demographic and medical variables were explored using ANOVA, followed by Bonferroni post-hoc tests, t-tests, χ2 for independence, Fisher’s exact test, and Kruskal–Wallis’s test in accordance with the variables’ scales. The relationships between EDs, suicidality, and self-injury were explored using ANOVA, followed by Bonferroni post-hoc tests. EDs, LA, and impulsivity were explored using ANOVA followed by Bonferroni post-hoc tests. Predictors of suicidality and self-injury among ED patients were discovered using Pearson correlations. Finally, linear and logistic regression analyses for the prediction of suicidality and self-injury among two groups of ED patients were conducted.

## Results

### Participant Characteristics

Participants in all study groups did not differ in terms of marital status, occupation, and age. Significant differences were found in level of education, psychiatric medication, psychotherapy, ADHD, and depression. As demonstrated in **Table 1**, participants from the control group were more educated, less depressed, and had a lower probability of ADHD, psychiatric medication, and psychotherapy compared to participants with EDs (p < .001). Diagnosis groups did not differ in terms of any of these variables, or in terms of other illness variables, besides BMI, which was higher among binge/purge patients compared to AN-R patients (p < .001).

**Table 1:** Sample description*.

Variable	AN-R (N = 25)	AN-BP/BN (N = 55)	Control (N = 37)	Group differences
Marital status				
Married	2 (8%)	6 (10.9%)	5 (13.5%)	FET = 1.76, p > .05
Single	23 (92%)	48 (87.3%)	32 (86.5%)	
Divorced	0 (0%)	1 (1.8%)	0 (0%)	
Education level				
High-school	10 (40%)	28 (50.9%)	0 (0%)	K-W χ^2^(2) = 18.83, p < .001
Higher edu.	10 (40%)	11 (20%)	22 (59.5%)	
Academic	2 (8%)	10 (18.2%)	14 (37.8%)	
MA	1 (4%)	3 (5.5%)	1 (2.7%)	
Other	2 (8%)	3 (5.5%)	0 (0%)	
Occupation				
Employed	12 (48%)	33 (60%)	24 (68.6%)	χ^2^(2) = 2.57, p > .05
Unemployed	13 (52%)	22 (40%)	11 (31.4%)	
Psychiatric medication				
Yes	12 (50%)	29 (54.7%)	2 (5.4%)	χ^2^(2) = 23.04, p < .001
No	12 (50%)	24 (45.3%)	35 (94.6%)	
Psychotherapy				
Yes	22 (88%)	53 (96.4%)	14 (37.8%)	χ^2^(2) = 44.11, p < .001
No	3 (12%)	2 (3.6%)	23 (62.2%)	
ADHD				
Yes	8 (32%)	19 (34.5%)	1 (2.7%)	χ^2^(2) = 13.46, p < .001
No	17 (68%)	36 (65.5%)	36 (97.3%)	
Age (years)	22.54 (4.43)	24.55 (5.81)	23.16 (2.96)	F(2,114) = 1.84, p > .05, η^2^ = .031
Disorder onset age (years)	18.14 (3.56)	20.02 (5.75)	–	t(70.45) = −1.77, p > .05
Hospitalizations number	1.40 (2.20)	1.30 (2.51)	–	t(77) = 0.18, p > .05
Chronicity	4.40 (4.34)	4.18 (5.35)	–	t(78) = 0.18, p > .05
BMI	16.07 (3.12)	20.09 (4.79)	–	t(68.09) = −4.48, p < .001
Hospitalization (days)	4.90 (3.34)	4.61 (5.65)	–	t(64) = 0.21, p > .05
PHQ-Depression	17.35 (7.05)	19.90 (4.70)	4.38 (2.48)	F(2,102) = 123.80, p < .001, η^2^ = .708

*Categorical variables are presented in frequencies (and percentages). Continuous variables are presented in means (and standard deviations).

### NSSI and Suicide

**Figure 1:**
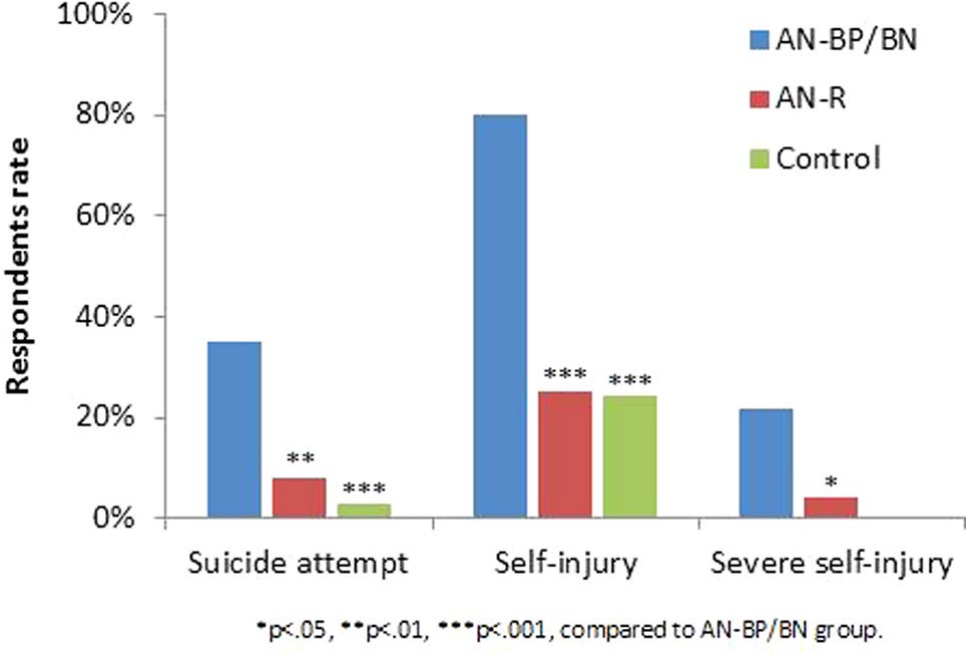
Suicide attempts, self-injury, and severe self-injury occurrence by group.

In line with our first hypothesis, differences between participants with different types of EDs (AN-R vs. binge/purge group) and participants without EDs were detected in all the suicidality and NSSI measures. The rate of participants who had attempted suicide was significantly different in all groups (FET = 17.22, p < .001): highest in the binge/purge group (34.8%), lower in the AN-R group (8%), and lowest in the control group (2.7%). Suicide ideation frequency was also significantly higher [F(2,114) = 33.44, p < .001, η2 = .370] in the binge/purge group (M = 2.04, SD = 1.41) compared to both the AN-R group (M = 0.64, SD = 0.99) and the controls (M = 0.22, SD = 0.63; p < .001). The rate of participants with self-injurious behavior was higher [χ2(2) = 35.53, p < .001] in the binge/purge group compared to both the AN-R and control groups. The rate of severe injuries was highest (FET = 12.16, p = .001) in the binge/purge group (21.8%), lower in the AN-R group (4%), and 0% in the control group. Self-injury frequency was higher [F(2,114) = 21.89, p < .001, η2 = .277) in the AN-BP/BN group (M = 3.26, SD = 2.12) compared to both the AN-R (M = 0.92, SD = 1.86) and control (M = 0.84, SD = 1.73) groups (p < .001) (**Figure 1**).

Differences between study groups in trait impulsivity were explored using ANOVA. Significant group effects were found in the attentional [F(2,114) = 14.34, p < .001, η2 = .201] and motor [F(2,114) = 9.72, p < .001, η2 = .146] factors, and in the total score [F(2,114) = 10.42, p < .001, η2 = .155] of the impulsivity questionnaire. Attentional and motor impulsivity scores, as well as the impulsivity total score, were higher among AN-BP/BN patients compared to AN-R patients and controls (p < .05), which did not differ from each other.

### Loss Aversion

**Figure 2:**
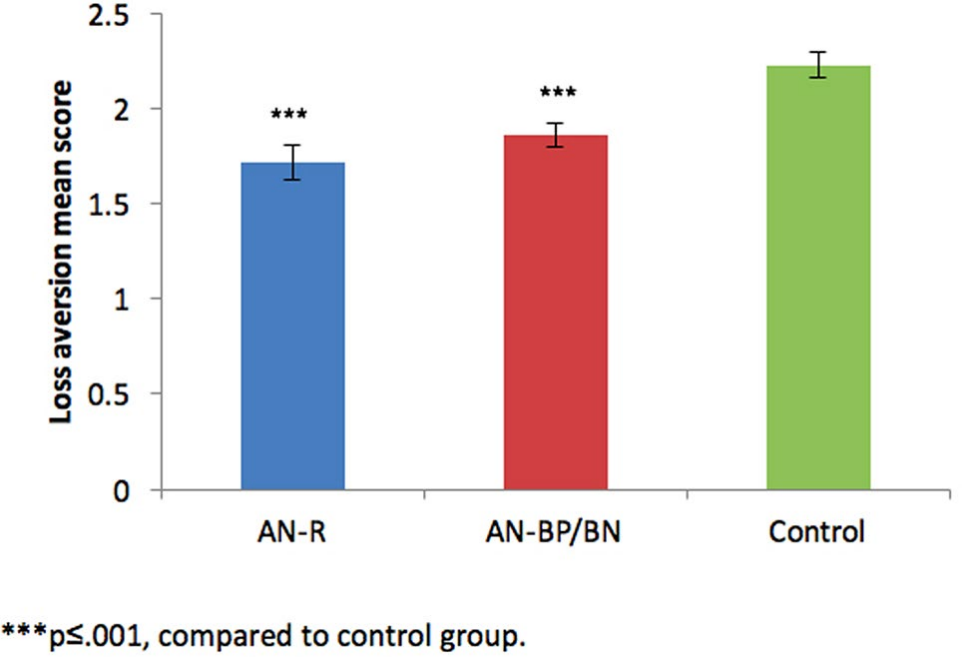
Loss aversion score by group.

Differences in LA between study groups were explored using ANOVA. Significant group effects were found in LA [F(2,111) = 11.41, p < .001, η2 = .171], while the Bonferroni post-hoc test showed lower LA scores among participants with EDs (AN-R or AN-BP/BN) compared to participants from the control group (p ≤ .001) as presented in **Figure 2**.

### Correlations Between Study Variables

To identify potential predictors of suicidality and self-injury, Pearson correlations between suicidality and NSSI and background variables, LA, and impulsivity were calculated.

As demonstrated in **Table 2**, older and unemployed participants reported higher frequency of suicide ideation, and an ADHD diagnosis was related to a higher probability of a suicide attempt. Higher BMI and depression scores were related to a higher probability of self-injury attempt, higher frequency of suicide ideation, and higher frequency of self-injury. Higher impulsivity scores were related to higher probabilities of suicide and self-injury attempts and higher frequency of suicide ideation. Interestingly and contrary to our hypothesis, LA scores were positively correlated with the probability of self-injury, frequency of self-injury, and frequency of suicide ideation.

**Table 2:** Correlations between suicidality and self-injury measures and potential predictors.

	Suicide attempt	Suicide ideation	Self-injury	Self-injury frequency
Age	.12	.24*	.05	.04
Work	−.07	−.31**	−.22	−.12
ADHD	.29**	.17	.09	.01
BMI	.18	.25*	.36***	.28*
PHQ-Depression	.21	.40***	.40***	.37**
Loss aversion	.01	.28*	.31**	.31**
BIS attentional	.30**	.31**	.38***	.21
BIS motor	.27*	.21	.32**	.17
BIS planning	.23*	.06	.18	−.05
BIS total	.31**	.22	.34**	.12

*p < .05, **p < .01, ***p ≤.001.

### Predictors of NSSI and Suicidal Behavior: Regression Analysis

Linear and logistic regressions were conducted for the prediction of suicide and NSSI variables in the ED group. Each analysis consisted of two steps. The first step included diagnostic and background variables, which were found to be correlated with the dependent variable. The second step included the LA and impulsivity variables. Taking into account multicollinearity considerations, only the BIS total score (not its subscales) was entered into the regression analysis. Diagnosis remained a significant predictor in all the analyses: an AN-BP/BN diagnosis predicted a higher probability of suicide and NSSI attempts, as well as higher frequencies of suicide ideation and NSSI compared to an AN-R diagnosis. An ADHD diagnosis contributed significantly to the prediction of suicide attempts, whereas the depression level explained other variances in NSSI attempts and frequency. Unemployment was recognized as another risk factor for suicide ideation. While impulsivity scores did not significantly contribute to the prediction of suicide and NSSI variables beyond the background measures, higher levels of LA were related to an increased risk for attempting NSSI, higher frequencies of NSSI, and suicide ideation (**Tables 3** and **4**).

**Table 3:** Logistic regression analysis for the prediction of suicide and self-injury attempts.

Step	Predictor	Suicide attempts			Self-injury		
		B (SE)	Wald	Odds ratio	B (SE)	Wald	Odds ratio
1	Diagnosis	1.91 (0.83)	5.48*	6.78	2.38 (0.82)	8.39**	10.79
	ADHD	1.47 (0.57)	6.69**	0.23			
	BMI				0.08 (0.10)	0.58	1.08
	PHQ				0.16 (0.07)	5.98*	1.18
	R^2^ = .24, χ^2^(2) = 14.25***				R2=.49, *χ*^2^(3)=27.56***		
2	Diagnosis	1.64 (0.84)	3.81*	5.13	3.42 (1.29)	7.03**	30.73
	ADHD	−1.29 (0.58)	4.91*	0.28			
	BMI				0.04 (0.11)	0.10	1.04
	PHQ				0.19 (0.08)	5.37*	1.22
	Loss aversion				3.74 (1.48)	6.35*	42.21
	BIS total	0.04 (0.02)	2.71	1.04	0.06 (0.03)	3.39	1.06
	R^2^ = .28, χ^2^_step_(1) = 2.91, χ^2^_model_(3) = 17.16***				R2=.67, *χ^2^*step(2)=13.96***, *χ^2^*model(5) = 41.52***		

*p ≤ .05, **p ≤ .01, ***p ≤ .001.

**Table 4:** Linear regression analysis for the prediction of suicide ideation and self-injury frequencies.

Step	Predictor	Suicide ideation		Self-injury frequency	
		B (SE)	β	B (SE)	β
1	Diagnosis	1.00 (0.37)	.32**	2.35 (0.58)	.47***
	Age	0.02 (0.03)	.07		
	Occupation	−0.71 (0.33)	−.25*		
	BMI	0.01 (0.03)	.05	−0.01 (0.05)	−.02
	PHQ	0.06 (0.03)	.23*	0.12 (0.04)	.29**
	*R^2^* = .35, F(5,59) = 6.31***			R2=.35, F(3,61)=10.97***	
2	Diagnosis	0.89 (0.36)	.29*	2.27 (0.56)	.45***
	Age	0.02 (0.03)	.09		
	Occupation	−0.70 (0.31)	−.24*		
	BMI	0.01 (0.03)	.03	−0.02 (0.05)	−.04
	PHQ	0.04 (0.03)	.17	0.11 (0.04)	.28*
	Loss aversion	0.72 (0.30)	.25*	1.21 (0.47)	.26*
	BIS attention	0.03 (0.03)	.09	−0.04 (0.05)	−.07
	*ΔR^2^* = .07, F(2,57) = 3.25*			ΔR2=.07, F(2,59)=3.52*	
	*R^2^* = .41, F(7,57) = 5.78***			R2=.42, F(5,59)=8.53***	

*p < .05, **p < .01, ***p < .001.

An additional set of regression analyses were conducted to determine whether the effect of the predictors on suicidality and self-injury differed between the diagnosis groups. While the regressions were identical to those presented in **Tables 3** and **4**, they also included an additional step of interactions between diagnosis and the other predictors. The interaction variables did not contribute significantly to the prediction of any of the dependent variables. Thus, the contributions of the abovementioned variables to the prediction of suicidality and self-injury seemed to be similar in both diagnosis groups.

To determine effect sizes that could be detected in this regression analysis, a *post hoc* power analysis was conducted using the software package, GPower (59). The sample size of 81 was used for the statistical power analyses and a eight predictor variable equation was used as a baseline. The recommended effect sizes used for this assessment were as follows: small (f2 = .02), medium (f2 = .15), and large (f2 = .35) (see 60). The alpha level used for this analysis was p < .05. The *post hoc* analyses revealed the statistical power for this study was.15 for detecting a small effect, whereas the power exceeded 0.82 and 0.99 for the detection of a moderate and large effect sizes, respectively. Thus, there was adequate power (i.e., power *.80) at the moderate to large effect size level, but less than adequate statistical power at the small effect size level.

## Discussion

### The Relationship Between Self-Harm and Impulsivity in EDs

Our first hypothesis addressed the differences in self-harm behavior and trait impulsivity between the various ED subgroups. In line with contemporary literature (7, 61–63), notable differences between the subgroups in self-harm behavior were found: individuals in the binge/purge group were more likely to demonstrate NSSI behaviors of a severer nature. Individuals in the binge/purge group also attempted suicide more often and were more preoccupied with suicide ideation compared to the AN-R group and healthy controls. The AN-R group had a higher rate of past attempts compared to the healthy controls, however, they were not significantly different from the controls in terms of NSSI prevalence or severity and suicide ideation. These results are in line with previous studies (30, 64, 65).

The trait impulsivity assessment points to a discrepancy between the ED subgroups. The binge/purge group scored higher on the impulsivity questionnaire, in general, and on the attentional and motor constructs of the BIS-11, in particular. These results support existing literature in the field (26, 65). AN-R patients did not differ from controls in trait impulsivity, thereby reinforcing previous findings which largely associate these patients with a compulsive cluster (21, 66).

### Is LA a Protective Mechanism?

Our second hypothesis regarding differences in LA between ED subgroups was partly confirmed. ED patients scored lower in LA than controls, suggesting that potential losses have a lower effect on the decisions of ED patients compared to controls. To the best of our knowledge, LA has not been linked to EDs prior to this study. The direction of the relationship corresponds with studies investigating impulsivity-prone populations, such as pathological gamblers and cocaine users, who have also been shown to display reduced levels of LA compared to healthy controls (67, 68). However, contradictory to our hypothesis, there were no differences in levels of LA between the AN-R and binge/purge subgroups (despite the difference in impulsivity). This may imply that decreased levels of LA are global in ED patients, and that whether characterized by either a loss of control or restraint, these individuals’ decisions tend to be less affected by potential negative outcomes compared to a healthy population.

### LA as a Predictor for Self-Harm Behaviors

Finally, we hypothesized a negative relationship between LA and self-harm behaviors among ED patients. Our reasoning was that those with high LA scores would be more likely to avert from self-harm due to its potential negative outcomes, such as physical injury. Conversely, as the decisions of patients with low scores in LA would be guided primarily by potential gains (i.e., release of anxiety, or end of suffering), we expected them to be more likely to engage in self-harm behaviors. Interestingly, the emerging results were the opposite of what we predicted: LA was positively correlated with the prevalence and severity of NSSI and with suicide ideation in the ED research group, while no significant association was found between past suicide attempts and LA. In addition, higher levels of LA were found to be a significant contributor to the prediction of NSSI behavior and frequency, and suicide ideation, over and above background variables and impulsivity. It is interesting to note that impulsivity was neither correlated with LA nor did it represent a unique contribution to the prediction of self-harm behaviors, perhaps due to overlap between this concept and other variables entered into the regression analysis.

How can this positive relation between LA and NSSI and suicidal ideation be explained? At face value, this finding contradicts recent findings according to which a high level of LA was shown to constitute a protective mechanism against suicidal behavior in a cross-sectional and longitudinal sample (42, 69). Two important factors may shed light on the causes for the contradictions in these findings: first, the distinction between non-suicidal self-injury and self-injury with an intent to die [see (70)], and second, differences between the populations studied in our and (69). Theorized that high levels of LA may avert the decision to attempt suicide due to its negative outcomes (e.g. death, pain to family members, etc.) superseding the positive outcomes associated with the act (putting an end to suffering). Individuals who display high levels of LA are therefore at a lower risk for carrying out an attempt. Compared to suicide, NSSI is a behavior that entails more moderate negative consequences (mainly physical pain and cosmetic damage), and perhaps less dramatic and somewhat advantageous consequences. Nock (33), for instance, suggests that although NSSI is considered a pathological behavior, it serves several intrapersonal and interpersonal purposes. Other studies have shown that a major intrapersonal gain of NSSI is affect regulation (71, 72). Another important positive aspect of NSSI is its communication-related function (73): studies concerning social media found NSSI to be driven by peer support (74, 75).

If we apply Hadlaczky et al.’s logic to NSSI, a decision-maker with a high level of LA should still be less likely to carry out NSSI than more so (as demonstrated by our data): even if the potential losses and gains are somewhat lower/different in NSSI, a high LA level should still play a preventive role. However, what if the decision is not related to carrying out or abstaining from NSSI? Given the high prevalence of suicidal ideation in the ED population, the decision may pertain to engaging in NSSI rather than carrying out a suicide attempt. In this case, participants with high LA levels may be redirected from the “bad” alternative of attempting suicide (with significant potential negative consequences) to the “less bad” alternative of carrying out a NSSI.

Taken together, the choice of NSSI as a better alternative over an intently driven suicide attempt relies not only on several predisposed variables, including hopelessness, depression, and the individual’s communication aptitude, but also on his or her ability to assess potential losses. For individuals in need of immediate affect regulation, a tendency toward higher LA may safeguard them from choosing a self-regulation behavior with a higher potential for a negative consequence (suicide attempt), while motivating them toward a communicative act. Thus, although a high level of LA is considered a protective factor against high risk decisions, it may serve as a facilitator of lower risk decisions that help the individual soothe and communicate his or her own suffering (76, 77). This explanation is congruent with the results of a study conducted by Gómez-Expósito et al. (78), which demonstrated that while bulimic-spectrum patients with past suicide attempts scored high on impulsivity, bulimic patients with NSSI presented lower impulsivity, most probably as a manifestation of a different mechanism of self-regulation. Models that determine the transition from NSSI to suicide attempts may support this hypothesis as well (79)—a growing sense of capability for self-harm may decrease levels of LA and lead the individual to choose a more lethal option. It is also possible that while LA remains constant, the option of suicide becomes increasingly suitable for patients whose suffering persists and intensifies.

Regarding the positive relation between higher levels of LA and the preoccupation with suicidal thoughts, further study is required. Research on suicide ideation has failed to discriminate between suicide attempters and non-attempters (80), thereby leading to the notion that it is an independent variable. Taking this a step forward, we suggest that suicide ideation is part of a repertoire of actions, which, under certain circumstances, may serve as a satisfactory alternative to the carrying out of an intentional suicide attempt. The understanding of the individual in pain that death is an alternative to suffering, can, to some extent, alleviate suffering and allow him or her to go on with their lives. This idea reinforces the notion offered in work presented by Hadlaczky et al. (69) proposing that LA can be found as an important variable in understanding the transition from suicidal thoughts to attempts. Further study in this field is required to confirm these results. It would also be interesting to investigate the relationship between LA and suicidal using other measures than the lottery task in this study, which has a quite specific economic/statistical character. An example could be to use for instance a “mug-task” type paradigm (81), and perhaps through the endowment effect investigate other domains such as the loss of relationships or status.

### Limitations

A major drawback of this study is its cross-sectional design, which prevents us from drawing conclusions regarding cause and effect. Additionally, considering difficulties in the recruitment of EDs patients, due to limited cooperation and medical complications, a relatively small sample size of participants (93) were recruited in the research group. This could explain why we did not find a direct LA effect on attempted suicide in our data, contradictory to previous studies (69), possibly due to a lack of statistical power. Moreover and in favor of this option, the number of attempted suicides in the data was relatively low (21 out of 81 subjects in the research group). In addition, future studies with a larger sample size might determine an interaction effect between the contributing variables and the diagnosis which was not found in this research.

Although all participants were recruited from the same medical center, some were patients already in treatment, while others were patients interviewed prior to treatment as a part of an intake procedure. This might affect the temporal state of the patients and result in alterations in the performance of the behavioral task. Due to the fact that some of the potential participants refused to participate we cannot rule out a sampling bias in our study sample. By including female participants only, compared to our study does not account for potential differences in coping mechanisms and behavioral tendencies attributed to gender differences, which were found to be significant (82). Further study that involves the participation of male subjects could help resolve this ambiguity.

## Data Availability Statement

The raw data supporting the conclusions of this manuscript will be made available by the authors, without undue reservation, to any qualified researcher.

## Ethics Statement

The studies involving human participants were reviewed and approved by IRB SHEBA Tel Hashomer. The patients/participants provided their written informed consent to participate in this study.

## Author Contributions

ES and YG planned and conducted the research, assessed the data, and wrote the paper. GH took a part in analysis of the data and contributed to the discussion, specifically in specialty areas of decision making paradigms. NS was responsible for data collection and contributed to analysis of the data. EG took a part in theoretical and practical aspects of research planning and data collection. NH was involved in the formulation of the initial research.

## Conflict of Interest

The authors declare that the research was conducted in the absence of any commercial or financial relationships that could be construed as a potential conflict of interest.
